# Quantitative and Qualitative Changes in the Genetic Diversity of Bacterial Communities in Anaerobic Bioreactors with the Diatomaceous Earth/Peat Cell Carrier

**DOI:** 10.3390/cells11162571

**Published:** 2022-08-18

**Authors:** Agnieszka A. Pilarska, Agnieszka Wolna-Maruwka, Alicja Niewiadomska, Jarosław Grządziel, Anna Gałązka, Emil Paluch, Klaudia Borowiak, Krzysztof Pilarski

**Affiliations:** 1 Department of Hydraulic and Sanitary Engineering, Poznań University of Life Sciences, ul. Piątkowska 94A, 60-649 Poznan, Poland; 2 Department of Soil Science and Microbiology, Poznań University of Life Sciences, ul. Szydłowska 50, 60-656 Poznan, Poland; 3 Department of Agriculture Microbiology, Institute of Soil Science and Plant Cultivation—State Research Institute, ul. Czartoryskich 8, 24-100 Pulawy, Poland; 4 Department of Microbiology, Faculty of Medicine, Wroclaw Medical University, Tytusa Chałubińskiego 4, 50-376 Wroclaw, Poland; 5 Department of Ecology and Environmental Protection, Poznań University of Life Sciences, Piątkowska 94 C, 60-649 Poznan, Poland; 6 Department of Biosystems Engineering, Poznań University of Life Sciences, ul. Wojska Polskiego 50, 60-627 Poznan, Poland

**Keywords:** microbial carrier, anaerobic bioreactors, genetic diversity, microbial adhesion, BioFlux system, microbiome visualisation

## Abstract

This paper analyses the impact of the diatomaceous earth/peat (DEP; 3:1) microbial carrier on changes in the bacterial microbiome and the development of biofilm in the anaerobic digestion (AD) of confectionery waste, combined with digested sewage sludge as inoculum. The physicochemical properties of the carrier material are presented, with particular focus on its morphological and dispersion characteristics, as well as adsorption and thermal properties. In this respect, the DEP system was found to be a suitable carrier for both mesophilic and thermophilic AD. The evaluation of quantitative and qualitative changes in the genetic diversity of bacterial communities, carried out using next-generation sequencing (NGS), showed that the material has a modifying effect on the bacterial microbiome. While Actinobacteria was the most abundant cluster in the WF-control sample (WF—waste wafers), Firmicutes was the dominant cluster in the digested samples without the carrier (WF-dig.; dig.—digested) and with the carrier (WF + DEP). The same was true for the count of Proteobacteria, which decreased twofold during biodegradation in favor of Synergistetes. The *Syntrophomonas* cluster was identified as the most abundant genus in the two samples, particularly in WF + DEP. This information was supplemented by observations of morphological features of microorganisms carried out using fluorescence microscopy. The biodegradation process itself had a significant impact on changes in the microbiome of samples taken from anaerobic bioreactors, reducing its biodiversity. As demonstrated by the results of this innovative method, namely the BioFlux microfluidic flow system, the decrease in the number of taxa in the digested samples and the addition of DEP contributed to the microbial adhesion in the microfluidic system and the formation of a stable biofilm.

## 1. Introduction

Nowadays, the anaerobic digestion (AD) process is seen as a reliable and integrated system for environmental protection and energy security, reducing problems associated with the accumulation of organic waste [1,2]. AD is a biotechnological process assisted by syntrophic microorganisms leading to the controlled bioconversion of organic waste into biomethane, which is then converted in cogenerators enabling the generation of electricity and heat. The AD process also provides a real opportunity to control emissions of volatile compounds and air pollutants. In principle, AD is a technology that strengthens closed-loop recycling management systems by reducing greenhouse gas emissions and producing value-added products for the economy and society [3,4]. However, anaerobic biodegradation is limited by three factors most commonly cited in the literature: (1) the limited rate of bioconversion at the substrate hydrolysis level, (2) low conversion rates of biomass, including key intermediate products such as propionic and butyric acids, which is caused, among other things, by (3) a too slow growth of anaerobic bacteria during methanogenesis [5]. According to the majority of researchers, the rate of cell proliferation in anaerobic bioreactors and their high metabolism improve biogas recovery at a high rate of treatment. The treatment capability of such a biological system is directly associated with the number of microorganisms effectively retained in the environment and enzymatic activity [6]. The genetic diversity of bacterial communities and the rate and direction of changes within the metapopulation are also important for the efficiency of the AD process [7].

Immobilised cell systems significantly improve the productivity of the bioreactor and protect the microorganisms involved in the biotechnological process from adverse environmental conditions. To enrich biogas with methane, researchers have so far used a variety of carrier materials, most of which are characterised by a well-developed specific surface area and an interesting pore structure with a significant pore volume [8]. These materials have a sufficient number of sites to attach microorganisms, thus ensuring their rapid growth and proliferation. Moreover, reactive groups on the surface of microorganisms can interact with the carrier material, which guarantees strong attachment and prevents biomass losses. Recent scientific reports confirm the efficiency of carriers in anaerobic digestion and various types of biocarriers, including biopolymers derived from renewable biomass sources (lignin, chitosan, polystyrene, polypropylene rings, sugar beet pectin), as well as traditional ones: zeolites, glass rings, ceramsite and magnetite [9,10,11].

An excellent solution used as a bacterial carrier in AD is the efficient diatomaceous earth/peat system; its physicochemical properties and positive impact on the efficiency of AD were presented in the authors’ recent publication [12]. Similar to other carriers used in AD, DEP has the required physicochemical and microstructural properties (including, inter alia, the above-mentioned considerable specific surface area, porosity, and mechanical and thermal stability), in addition to being non-toxic and widely available. As a result, it can be used on an industrial scale.

The use of AD monitoring strategies using conventional physicochemical parameters has greatly facilitated the implementation of this technology in industrial-scale applications [13]. However, the above-mentioned parameters only reflect the current status of the process and provide information on the microbial community composition, dynamics, and activity, which directly determines the efficiency of the process [14]. For this reason, research on the interaction between the temporal trajectories of microbial community structure and operational parameters is systematically carried out in numerous research centres all over the world. The implementation of high-throughput sequencing techniques in AD research significantly increased our knowledge of the functioning of microbial communities [15,16]. Amplicon sequencing made it possible to identify Actinobacteria, Bacteroidetes, Chloroflexi, Firmicutes and Proteobacteria as dominant bacterial phyla and helped reveal several acetoclastic and hydrogenotrophic methanogens. Acetoclastic *Methanosaeta* was mostly observed in the digested medium under stable process conditions, while a transition to hydrogenotrophic methanogenesis often occurred under deteriorated conditions, e.g., increased salinity and/or total ammonia nitrogen (TAN) concentration [17,18]. The application of ‘omics’ techniques also helped to identify genes relevant to carbohydrate, lipid and protein metabolism in AD [19].

The next-generation sequencing (NGS) analysis conducted during the previous study by Pilarska et al. (2021) revealed the presence of twelve unique bacteria in objects with the addition of the silica/lignin carrier (4:1), including the genera of *Bacillus*, *Ligilactobacillus*, *Micromonospora*, *Microbacterium*, and *Staphylococcus* [7]. The researchers recorded the highest values of Simpson’s and Shannon’s biodiversity coefficients for both waste wafer and cheese co-substrate control systems, WFC (without the carrier) and for the WFC samples with the carrier. It was also proved that the research objects, composed of stabilised sewage sludge as inoculum, were dominated by three types of bacteria such as spore forming Firmicutes, Proteobacteria and Actinobacteria; as shown by the literature reports, the first two dominated in the AD environment of sewage sludge [20]. According to the literature, many of the known syntrophic acetate-oxidising bacteria are Firmicutes. This type also includes a large group of microorganisms that play an important role in the degradation of volatile fatty acids and in the digestion of polysaccharides, oligosaccharides and proteins, including, for example, a genus of Gram-positive anaerobic *Clostridium* bacteria [21].

The aim of this study is to evaluate the impact of a new diatomaceous earth/peat AD cell carrier on the genetic diversity, survival and morphological characteristics of bacterial communities, as well as the intensity of biofilm formation—in the anaerobic digestion process. The state-of-the-art analytical techniques such as, in particular, next-generation sequencing (NGS), the BioFlux microfluidic flow system, and fluorescence microscopy were used. The authors investigated the physicochemical properties of the carrier material, including mainly morphological, dissipation and adsorption properties, the dynamics of changes in the number of anaerobic bacteria during the process, changes in the structure of the bacterial microbiome of samples collected from anaerobic bioreactors, the ability of the analysed samples to adhere and form a biofilm over time, as well as the morphology and the degree of differentiation of spatial forms of the biofilm. This article is a continuation of the paper published by Pilarska et al. (2022) on the impact of DEP on the stability and efficiency of the AD process of selected food waste [12].

## 2. Materials and Methods

### 2.1. Feedstock and Carrier Material

The experiment was carried out with waste wafers with filling, provided by a production company based in the Wielkopolskie Voivodeship (Wolności, Poland), as feedstock. Stabilised sewage sludge (SS) delivered by the sewage treatment plant in the city of Poznań (Poland) was used as inoculum. The microbial carrier was fed to the bioreactor under anaerobic conditions in the following doses per 1 L of the batch: diatomaceous earth, (Perma-Guard, Inc., Bountiful, UT, USA), 15 g and deacidified peat (Torf Corporation Sp. z o.o., Wrocław, Poland), 3 g—diatomaceous earth/peat, DEP 3:1 [12]. Hybrid adhesive bonds between the two components were formed during mechanical grinding carried out with the use of a ball mill (PULVERISETTE 23, FRITSCH, Fellbach, Germany). The carrier was then washed with PBS (phosphate-buffered saline) solution, treated with sterile distilled water and dried at 70 °C.

### 2.2. Batch Anaerobic Digestion

The anaerobic digestion process was carried out in relation to two samples (as two batches): a control sample, WF-control and a sample with the carrier, WF + DEP (the laboratory in the Poznań University of Life Sciences). The ratio of feedstock to inoculum in batches, where the content of total solids (TS) did not exceed 10%, was determined according to the recommendations of the German VDI 4630 standard [22]. The composition and basic parameters of the samples are shown in Table 1.

The anaerobic digestion (AD) process was carried out in a multi-chamber bioreactor, the diagram of which was presented in the authors’ previous publication on the impact of the DEP carrier on the biogas efficiency of digested waste [12]. The bioreactors with a capacity of 1.0 L were filled with the feedstock, which was kept under anaerobic conditions and stirred once a day. Six biofermenters (each sample was fermented three times) were placed in a water jacket connected to a heater, which allowed the process to be carried out at a set range of temperatures (mesophilic conditions, 39 °C). The produced biogas was directed into tanks (with a scale) where it was stored. The hydraulic retention time (HRT) of the fermentation process was 21 days. As per the German standard DIN Guideline 38 414-S8 (DIN, Deutsches Institut für Normung, Köln, Gemany) [23], the experiment was performed until the daily production of biogas dropped below 1% of the total biogas produced, in all biofermenters. The amount of biogas produced was checked every 24 h. The samples of sludge for microbiological tests, the results of which are the focus of this paper, were taken (1) just after loading the batch, before the addition of the carrier, WF-control, and at the last stage of the process: (2) WF-dig. (dig.—digested) and (3) WF + DEP. The WF-control sample is therefore a reference material for changes in the microbiome of the digested material, both without the carrier (WF-dig. sample) and with the addition of the carrier (WF + DEP sample). 

### 2.3. Physicochemical Analysis 

The feedstock, inoculum and prepared batches, were subjected to physicochemical analysis. The methodology of the analysis performed and the obtained parameter values are presented in the authors’ previous publication [12].

The cell carrier was also subjected to physicochemical analysis (morphological-dispersive characteristics and porous structure properties), the methodology of which was described by the authors in an earlier articles [3,7].

### 2.4. Analysis of the Total Bacterial Count

Analyses performed with the use of selective standard agar by Merck (Darmstadt, Germany) made it possible to measure colony-forming units (CFUs) of anaerobic bacteria (AnB). The bacterial count was recorded after 24 h of incubation at 35 °C [7]. Anaerobic conditions under which Petri dishes were incubated were maintained using the Anaerocult anaerobic incubation system (Merck, Darmstadt, Germany). 

### 2.5. DNA Extraction and Next-Generation Sequencing (NGS) 

Total DNA was extracted from 500 mg of each sample using Genomic Mini AX Soil kit (A&A Biotechnology, Gdynia, Poland) according to the manufacturer’s instruction. The extracted DNA was quantified using Quant-iT HS ds.-DNA assay kit (Invitrogen, Carlsbad, CA, USA) on a Qubit2 fluorometer (Invitrogen); 2 µL of extracts were examined with the use of a 0.8% agarose gel. Metagenomic analysis was conducted based on the hypervariable region V3–V4 of the 16S rRNA gene. Specific primers 341F and 785R were used for amplification of this region and library preparation. PCR (polymerase chain reaction) was conducted with the use of Q5 Hot Start High-Fidelity DNA Polymerase kit (NEB Inc., Ipswich, MA, USA) at reaction conditions according to the manufacturer’s specifications. Sequencing was conducted with the use of a MiSeq sequencer in 2 × 250 bp paired-end (PE) technology, 14, 4429 7 of 23 using the v2 Illumina chemistry kit [7]. The reactions were carried out according to the Illumina V3–V4 16S RNA amplification protocol (Illumina, Inc., San Diego, CA, USA) and sequencing was performed according to the Illumina MiSeq PE300 (Genomed S.A., Warsaw, Poland). Automatic data analysis was performed on MiSeq and in Cloud environment BaseSpace by Illumina, using the 16S Metagenomics protocol (ver. 1.0.1). The libraries were prepared in an analogous way, according to the attached Illumina protocol. 

### 2.6. Bioinformatics and Statistical Analysis

Demultiplexed fastq files were processed using the *DADA2* (1.14) package [24] in R software (3.6.0) [25]. Based on the quality plots, the last 20 and 70 bases were trimmed off forward and reverse reads accordingly. Primer sequences were excluded from all reads. Filter parameters were as follows: *maxN* = 0, *maxEE* for both reads = 2, *truncQ* = 2. The error rates were estimated by *learnErrors* using one million reads and exact sequence variants were resolved using *dada*. Then, *removeBimeraDenovo* was used to remove chimeric sequences. After the filtration steps, 81,729–112,956 (mean = 96,300) of the reads were kept for further analysis. Taxonomy was assigned against the latest version of the modified RDP (Ribosomal Database Project) v18 database [26] using IDTAXA [27] on the sequences table, which was the outcome of the *DADA2* workflow described above. Then, the results were converted and imported into the *phyloseq* (1.22.3) package [28]. Sequences that belonged to the chloroplast or mitochondrial DNA were excluded. Then, for the purposes of further analysis, the total number of reads of individual taxa was converted into a percentage, assuming the sum of all taxa in individual samples to be 100%. On average, 61% of all reads, which were correctly classified to the genus level, were aggregated and counted. Unclassified reads were grouped using *vsearch* [29] implemented in the seed software, version 2.1 [30] at a 99% similarity level. Each of 65 clustered groups of unclassified reads was then marked as Unclassified_001 to Unclassified_065 and merged with the previous table (containing reads classified to the genus level). This approach enabled the statistical processing of the true alpha and beta diversity, regardless of whether a sequence exists in the reference database or not. In total, 183 unique taxa (at the genus level plus unclassified clusters) were detected in all samples. Then, 11 out of the most abundant sequences unclassified to the genus level were selected and analysed using the BLAST (Basic Local Alignment Search Tool) tool (NCBI) to search for the closest relative bacteria. The phylogenetic tree was generated using the online iTOL (Interactive Tree Of Life) tool [31].

### 2.7. BioFlux Microfluidic Flow System 

The BioFlux 1000 microfluidic flow system, with 48-well plates (Fluxion, San Francisco, CA, USA), was used to study adhesion and biofilm formation in a continuous flow. The samples were prepared in the following way: for each sample (firstly: for WF-control and at the last stage of AD: for WF-dig. and WF + DEP), 1.5 mL of the sediment was collected from the bioreactor. The supernatant was then centrifuged (5000 RPM, 5 min), retained and used as a culture medium. Standardised 0.5 OD test suspensions were prepared by taking 100 µL of the raw sample and suspending it in an appropriate volume of the aforementioned supernatant. Initially, the inoculum (1 mL) was incubated without flow for the adhesion of samples, the wells were filled, and the flow was initiated at 0.5 dyn per 1 cm^2^. The plate was incubated at 28 °C for 24 h in the incubator chamber (Carl Zeiss Pecon Incubator XL S1, Erbach, Germany). In the environmental chamber, in order to obtain the closest optimal anaerobic conditions during incubation, the gas compositions recommended for the anaerobic chamber (nitrogen 80% and carbon dioxide 20%) were used. Parameters such as flow shear, adhesion time, and inoculum concentration were tested for the production of biofilm in the microfluidic system. Imaging was performed at 1 h intervals using an Axio Inverted Observer fluorescence microscope 7 (Carl Zeiss, Erbach, Germany) equipped with an Orca Flash 40 camera (Hamamatsu, Hamamatsu-city, Japan) and 20× objective (differential interference contrast (DIC) mode). Scale bar = 100 µm. Microscopic images from the experiments were analysed using the Bioflux Montage software (Fluxion, San Francisco, CA, USA) integrated with the system [32]. The tests were performed in three biological replicates with three technical replicates constituting three different fragments of microcapillaries.

### 2.8. Visualisation of Microbiome Using Fluorescence Microscopy

Direct microscopic preparations of bioreactor samples were made. For samples (WF-control, WF-dig. and WF + DEP), they were prepared by adding 100 µL of each bioreactor sample to 900 µL of PBS with fluorescence dyes (1 µL Syto 9 (Ex λ = 488 nm) and 1 µL of propidium iodide (Ex λ = 543 nm) (PI). A DAPI (4′,6-diamidino-2-phenylindole) filter set (Ex λ = 359 nm) was also used in the visualisation to excite the autofluorescence of the carriers. This iridian single band filter set consists of the 377-60 EX, 447-60 EM and 409 DM filters. It is optimised for 4′,6-diamidino-2-phenylindole, DAPI and other fluorophores such as Alexa Fluor 350, AMCA, BFP, Hoechst 33258, Hoechst 33342, Hoechst 34580, LysoSensor Blue, Marina Blue, Pacific Blue and sgBFP. The samples were incubated for 30 min under anaerobic conditions and then quickly visualised by making microscopic preparations using two basic glasses [33]. The samples were analysed using fluorescent microscopy (magnification: 10× and 40×) by means of an Olympus BX51 + DP25 camera (Olympus, Japan). Software CellSens Dimension Olympus. Scale bar A = 100 µm and B = 20 µm.

## 3. Results and Discussion

### 3.1. Characterisation of the Cell Carrier

The carrier used in this experiment meets the requirements of a good cell matrix. Due to its specific microstructure, mainly determined by the high proportion of diatomite, the material is characterised by high adhesion to microorganisms [34]. The presence of peat in the system, with its highly porous and rough microstructure, is also important for the formation of biofilm. Apart from increasing the adhesion of the matrix to cells, peat also contributes to the improvement of the absorption capacity of the matrix and, due to its chemical composition, including the mineral nutrients it contains, acts as a medium [35]. The positive results of the enzymatic activity analysis and the enhanced bacterial multiplication dynamics during the process, as presented in the following Section 3.2, are a clear effect of the addition.

**Figure 1:**
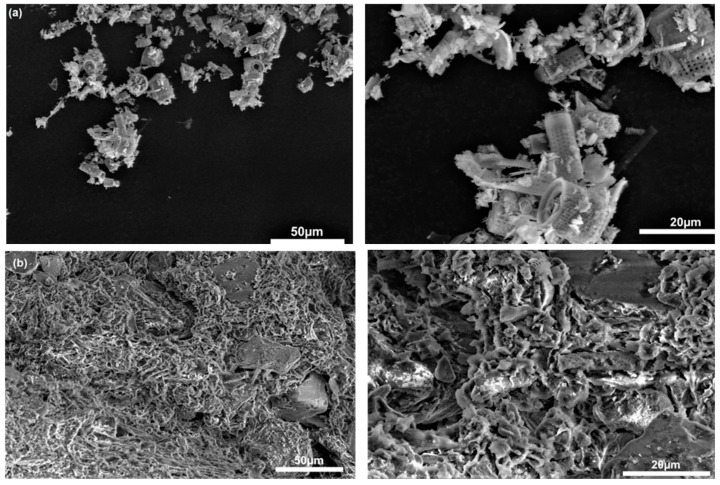
SEM images of (**a**) diatomaceous earth (**b**) peat at different magnifications: 50 and 20 µm diameter particles.

The SEM images (see Figure 1a,b; magnification 20 and 50 µm) show the morphological and microstructural properties of diatomaceous earth (a) and peat (b). Microscopic images of diatomite present cylindrical elements with regularly spaced holes, which makes them look like a honeycomb [36]. This issue is discussed in detail in the authors’ recent publication [12]. It should also be noted that some of the SEM images presented in the literature, reveal a contaminated surface of raw diatomaceous earth with partially blocked pores. Their specific distribution in the material is also emphasised: macropores are observed in the middle area of the diatomite disk and mesopores—in the peripheral area. Such a pore structure intensifies adsorption processes. The SEM images of the second component of the carrier, namely peat, are shown in Figure 1b. This is a microscopic image of powder, obtained by drying and grinding peat. Its irregular microstructure, observed in the image, confirms the results presented in other publications [37].

The dispersion of powders also affects the microstructural form [12]. According to literature data, the dominant value of the diameter of particles in a sample of raw diatomite is around 34 µm [38]. Information on the dispersion of peat was also presented by Bartaczak et al., 2015 [39], using a graph of particle size distribution by volume fraction. The average diameter of peat particles, D in the volume of the peat sample analysed by the author [40] was 49.1 µm. The analysis of the particle size distribution in diatomite and peat confirmed the tendency of both diatomite and peat to form aggregates and agglomerates of particles, which significantly shapes the development of the specific surface of the materials.

**Figure 2:**
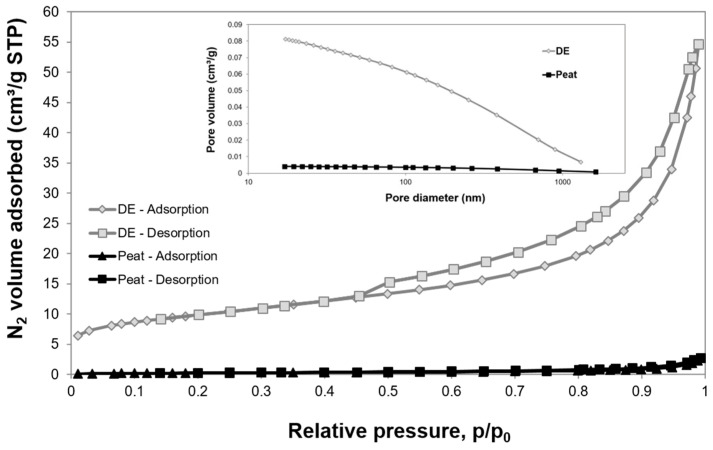
N_2_ adsorption/desorption isotherms and pore size distribution of the diatomaceous earth (DE) and peat.

The size of the BET specific surface area, along with the porosity, stability, and non-toxicity of the microbial carrier, is one of the key parameters affecting the development of biofilm and the activity and survival of microorganisms in the biotechnological process. As shown in the study carried out by the authors [12], diatomaceous earth (DE) has a significantly developed surface area of 36.01 m^2^ g^−1^ with a pore diameter of 11.55 nm, whereas peat (P) is characterised by a much lower surface area of 1.20 m^2^ g^−1^ with a pore diameter of 18.83 nm. However, the values of the microstructural parameters of the DEP carrier material are similar to those obtained for pure diatomite [12]. Figure 2 presents nitrogen adsorption/desorption isotherms and the pore size distribution of the diatomaceous earth (DE) and peat. The volume of N_2_ adsorbed by the diatomite was much higher than that for peat and amounted to ca. 55 cm^3^ g^−1^ STP, while for peat it was only 2.7 cm^3^ g^−1^ STP (standard temperature and pressure).

**Figure 3:**
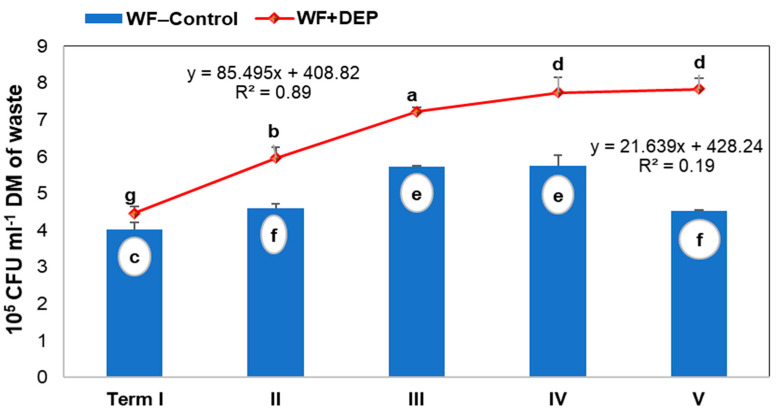
Bacteria total count changes found in the digested sampled material. Explanation: The same letter indicates a lack of significant differences (*p* < 0.05).

The rapid increase in the volume of gas, shown in Figure 2, occurred above the relative pressure p/p_0_ value of 0.8. Tsai et al. (2004) [41] observed the same behaviour in the case of diatomaceous earth by using alkaline activation. In contrast, in the case of the acid treatment used by Benkacem et al. (2016) [36], it can be observed that as the acid concentration increases up to 3 M, the adsorption-desorption isotherms shift towards a larger volume of adsorbed nitrogen for each partial pressure. This phenomenon was correlated with an increase in the specific surface area following an increase in the acid concentration. It appears that BET increases from 24 m^2^ g^−1^—for raw dolomite to 39 m^2^ g^−1^—after 3 M HNO_3_ treatment. The average value of the results obtained for the specific surface area of BET by Benkacem and his team [36] was similar to that of the amorphous calcined diatomite sample used in the present study (see Figure 3).

The adhesive bond between diatomite and peat leads to the formation of a thermally stable hybrid as demonstrated by the authors [12]. As shown in the literature, diatomaceous earth (loss of physically bound water and clay impurities—above 100 °C), which is the dominant component of the carrier, and peat (mass loss from ca. 220 °C), do not decompose in the temperature range corresponding to both mesophilic and thermophilic anaerobic digestion and can, therefore, act as a cell carrier in this process [42].

The chemical composition of both components of the DEP carrier system, the results of its elemental analysis and Fourier transform infrared spectroscopy (FT-IR) analysis have been presented in detail in the authors’ recent paper [12].

### 3.2. Total Bacterial Count in Digested Samples

Statistical analysis revealed that the addition of DEP carrier to food waste, subjected to an anaerobic digestion process, significantly affected the abundance of anaerobic microorganisms (Figure 3).

These conclusions correlate with the enzymatic activity results presented in the authors’ earlier publication [12]. According to Pilarska et al. (2021), silica, which is the basic component of diatomite, is a suitable microbial carrier due to its specific physicochemical properties [7]. In addition to the porosity and developed specific surface area mentioned in the previous chapter, it is characterised by a lack of toxicity to living organisms [43]. Additionally, the adsorption properties of peat have made it widely used for many years as a carrier for microorganisms included in various biopreparations. 

The immobilisation of microorganism cells on carriers contributes to increasing their density per unit volume of the fermenter, leading to better substrate utilisation and thus higher process efficiency and productivity [3]. Stalin and Prabhu (2007) proved in their paper that the addition of carrier to fermented animal manure contributes not only to an increase in microbial biomass but also to an increase in biogas generation efficiency [40].

The results also showed a significant effect of sampling date on the proliferation of anaerobic bacteria (see Figure 3). In the control variant, the abundance of the microorganisms analysed increased until date IV, after which a decrease was recorded. However, in the object with the addition of diatomaceous earth and peat (WF + DEP), the bacterial proliferation gradually increased until the end of the experiment. This means that as immobilised cells, with greater access to the medium throughout the process, they had greater survival and activity.

### 3.3. Bacterial Community Abundance and Composition

In addition to traditional methods of culturing bacteria, which were used to indicate the dynamics of changes in their abundance during the fermentation process of confectionery waste (Figure 3), a qualitative assessment of the present microbiome was also carried out using the increasingly popular and highly sensitive method of determining differences and similarities of microorganisms in a given environment; next-generation sequencing, NGS [7].

Sludge samples, which were subjected to NGS analysis, were taken before the addition of DEP carrier (WF-control) and in the last phase of AD (WF-dig., WF + DEP). The qualitative and quantitative analysis of the bacterial microbiome of the samples, as well as the in-depth comparative analysis discussed in this chapter, provides key information on the degree of modification of the bacterial microbiome in the course of a stable anaerobic biodegradation process (WF-dig.), and the same process with the participation of material supporting the process biocatalyst (WF + DEP).

**Figure 4:**
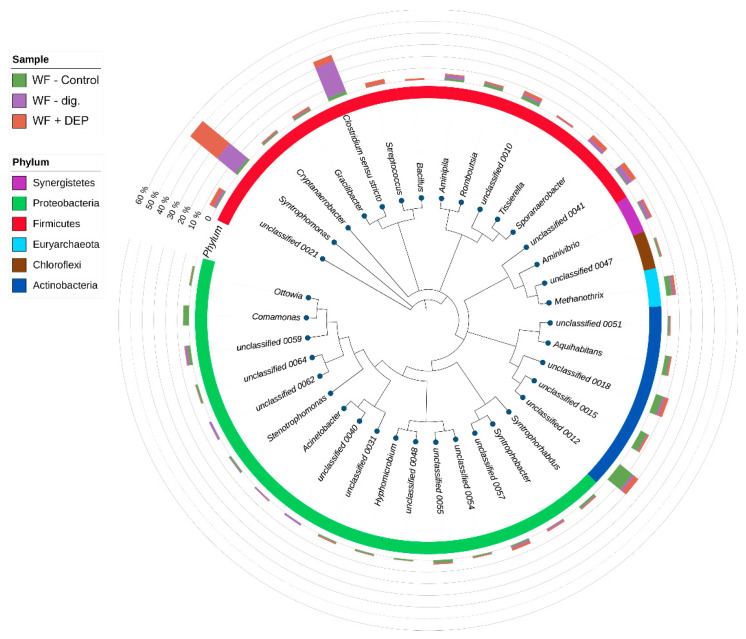
The phylogenetic tree representing the most abundant taxa (at the genus level). Outer bars visualise each taxa abundance in the corresponding sample. Inner bars visualise phylum affiliation of each genus.

Out of the 17 identified phyla, only six represent the dominant microbiota which constitutes 95–98% of all bacteria: Actinobacteria, Synergistetes, Proteobacteria, Firmicutes, Euryarchaeota, Chloroflexi (see the diagram below, Figure 4). The proportions of each phylum shifted by the end of the experiment, as most abundant phylum in sample WF-control was Actinobacteria (35%), its abundance decreased to 6.5% (WF-dig.) and 14% (WF + DEP). Simultaneously, Firmicutes became the dominant phylum (WF-dig., 68%; WF + DEP, 66%) compared to the control sample (WF-control, 21%). Similarly, the proportion of Synergistetes increased more than three and two-fold for WF-dig. and WF + DEP, respectively, while the abundance of Proteobacteria decreased about two-fold in the same samples.

At the genus level, *Syntrophomonas*, a bacterium known for its beta-oxidizing of saturated fatty acids and acetogenesis [44] was identified as the most abundant genus in two samples (WF-dig., 20%; WF + DEP, 30%) accounting for only 2 percent of WF-control. As previously shown by AD studies using a silica/lignin carrier (4:1), the *Syntrophomonas* genus is indeed predominant in the fermented confectionery (wafer) waste systems [7].

The low amount of *Syntrophomonas* in WF-control most likely results from the fact that the sample was essentially collected before fermentation; the increase in *Syntrophomonas* occurred, as expected, during the fermentation process (see WF-dig.) and the addition of DEP may have contributed to the increase in these bacteria by the recorded 10%. The addition of DEP seems to have an inhibitory effect on the growth of *Clostridium sensu stricto*. In the WF + DEP sample its abundance was twice as high as in the control sample (WF-control), while in the WF-dig. it was increased over nine-fold.

It is worth mentioning that despite the lack of unambiguous classification against the reference database, many unclassified sequences were also discovered systematically in different types of bioreactors by other researchers. This includes, among others unclassified_0041 which was found e.g., in a dark fermentation reactor by Chatellard et al. (2016), whereas in the study presented here, its abundance increased four times during fermentation compared to the control sample [45]. The number of unclassified_0021 at the endpoint was more than four-fold and two-fold higher, respectively, with and without DEP addition; this is consistent with other studies in which identical sequences were found e.g., in sludge from full scale anaerobic digester and bioreactors (see Table 2).

**Figure 5:**
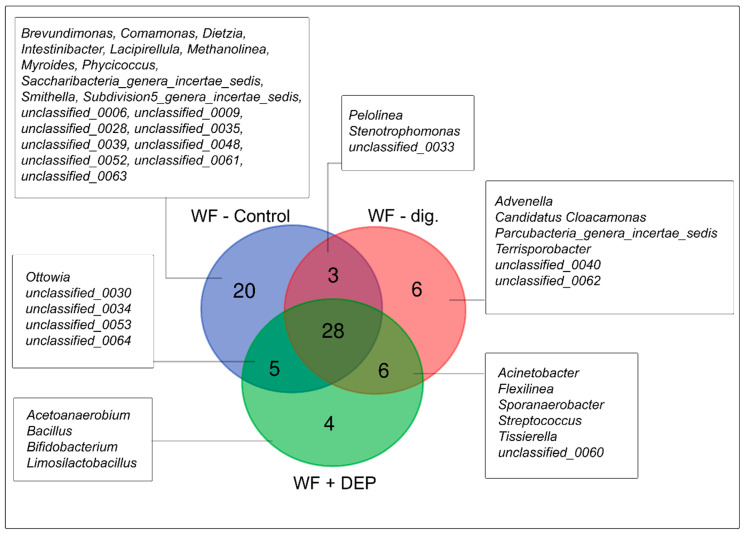
Venn diagram of overlapping bacterial communities from the three variants.

The meta-taxonomic analysis showed that the type of experimental facility influenced the structure of the bacterial microbiome, which is confirmed by the results as presented in a Venn diagram (see Figure 5).

Taking into account the presence of all taxa, 28 taxa common to all objects were selected within the genus. The highest number of unique taxa (20) was found in the WF-control variant. These included the genus *Brevundimonas*, *Lacipirellula*, *Myroides*, *Phycicoccus*, as well as the oxidase-positive motile gram-negative bacilli commonly found in the natural environment, capable of reducing nitrate to nitrite, belonging to the genus *Comamonas* [55]. Methane-producing archaeons of the genus *Methanolinea* have also been reported in the present experimental variant, in addition to the presence of the anaerobic propionate-degrading synthrophs *Smithella* sp. [56].

It was also shown that in the case of fermented waste, the duration of the process contributed to microbial succession. In the final stage of fermentation in the WF-dig. variant, two unique, unclassified taxa and four belonging to the genera *Advenella*, *Candidatus*, *Parucbacteria* and *Terrisporobacter* were recorded. In addition, the proportions of three types of bacteria, isolated from the waste at the commencement of the AD process (WF-control) as well as at its end (WF-dig.), were observed. These included: (i) a so far unclassified bacterial genus, designated in the study as unclassified_0033, (ii) anaerobic filamentous bacteria *Pelolinea* sp., included in the methanogenic microbiome community, and (iii) gram-negative bacteria of the genus *Stenotrophomonas*, which includes both saprophytic species commonly found in soil and opportunistic human pathogens [57].

The results of the NGS analysis also proved that the addition of the DEP system contributes to the modification of the bacterial microbiome. The experimental variant with addition of carrier, the lowest number of unique bacterial taxa was recorded, such as gram-positive *Bacillus* and *Bifidobacterium*; in addition, the presence of anaerobic and acetanogenic bacteria, belonging to the genus *Acetoanaerobium*, and heterofermentative lactic bacteria of the genus *Limosilactobacillus*, capable of producing exopolysaccharides, mostly present in the form of mucus, was recorded [58]. This effect confirms the selectivity of the carrier used in the process.

**Figure 6:**
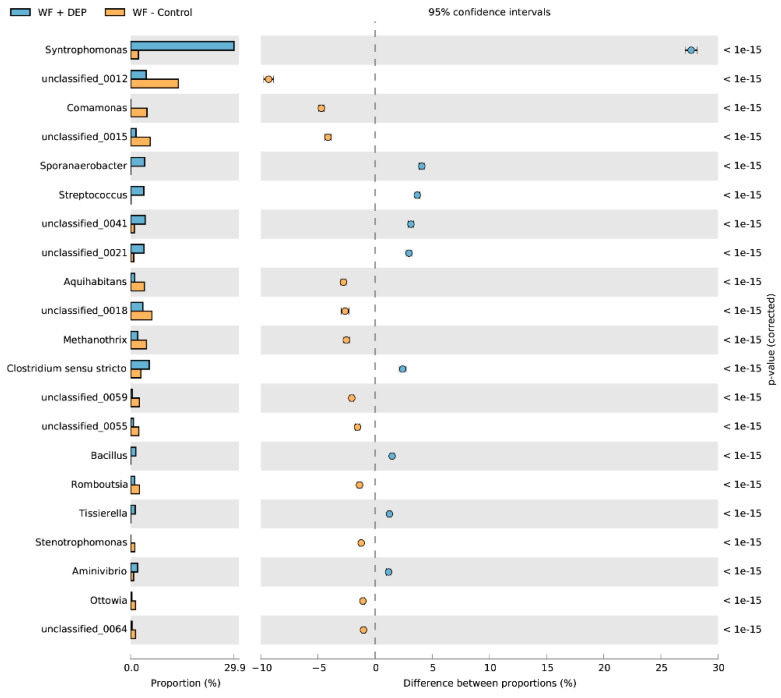
Comparison of bacteria genus composition between WF + DEP and WF-control variants.

**Figure 7:**
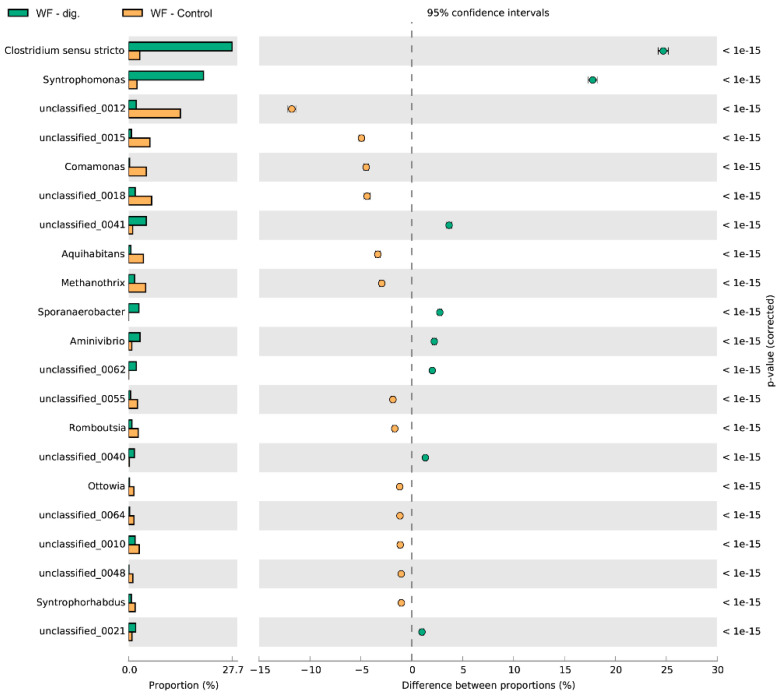
Comparison of bacteria genus composition between WF + control and WF + dig. variants.

**Figure 8:**
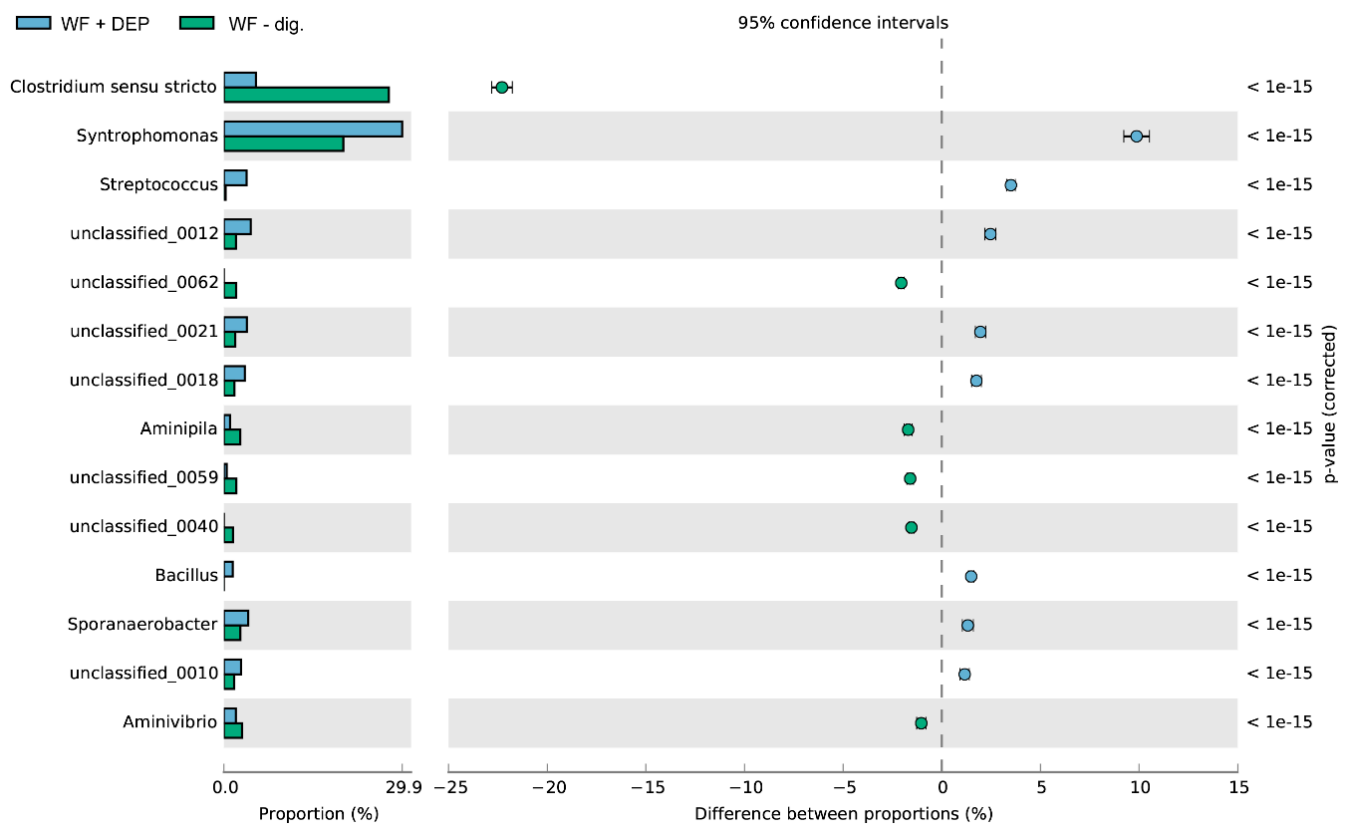
Comparison of bacteria genus composition between WF + DEP and WF-dig. variants.

The relative abundance of dominant bacterial genera, in each experimental system, expressed as a percentage of the sequence, based on NGS is presented in Figure 6, Figure 7 and Figure 8. Among the dominant taxa, the genera *Syntrophomonas*, *Comamonas*, *Aminivibrio*, *Sporanaerobacter*, *Streptococcus, Aquihabitans*, *Methanothrix*, *Clostridum*, *Bacillus*, *Romboutsia*, *Tissierella*, *Aminivibrio*, *Ottowia* and *Syntrophorhabdus* were identified.

Many unidentified bacterial sequences were also shown, demonstrating that research at this level should be carried out by sequencing longer DNA fragments. Studies by Campanaro et al. (2019) [59] and Theuerl et al. (2020) [60] indicate that the microbiome of waste, subjected to anaerobic digestion, can be variable and depends on the conditions in the bioreactor. Despite the many metagenomic analyses carried out, it is not strictly specified, so it will be extremely important to create a global genome database during future research. Nevertheless, according to the above-mentioned authors, the structure of the bacterial community of the ferment is extremely flexible, which allows it to quickly adapt to a wide range of temperatures and different types of substrates subjected to the fermentation process.

On the basis of the obtained results, significant statistical differences were observed in the structure of bacteria and their numbers at the genus level between the ferment without and with the addition of DEP carrier. It was found that apart from *Clostridium sensu stricto*, the genus of *Syntrophomonas* dominated in the analysed experimental variants. The highest percentage of *Syntrophomonas* sequences were recorded in the object with the addition of the carrier (WF + DEP) (see Figure 6 and Figure 7). It was also observed that the process of anaerobic degradation itself contributes to the multiplication of the present bacteria, as evidenced by the almost 80% increase in their numbers in the WF-dig. variant, as compared to WF-control (Figure 4 and Figure 7). Additionally, the study carried out by other authors shows that acetogenic bacteria of the genus of *Syntrophomonas*, which play an important role in the syntrophic oxidation of LCFA (long chain fatty acids), are one of the dominant genera in the anaerobic digestion of organic waste [61].

The metagenomic analysis of the fermented food waste carried out during the process also showed, irrespective of the type of experimental variant, a reduction in the sequence content of several unclassified bacterial genera and gram-negative, aerobic bacteria of the genus of *Comamonas* (Figure 8)*,* which contain lignin-oxidising multicopper oxidase. During the methane fermentation process, there was also a reduction in the abundance of oxidase- and catalase-positive bacteria of the genus of *Aquihabitans*, in addition to *Ottowia* bacteria, methanogenic bacteria *Methanothrix* sp. and hydrogen-producing bacteria of the genus of *Romboutsia* [62,63,64].

### 3.4. Adherence and Biofilm Formation Ability of Microorganisms 

**Figure 9:**
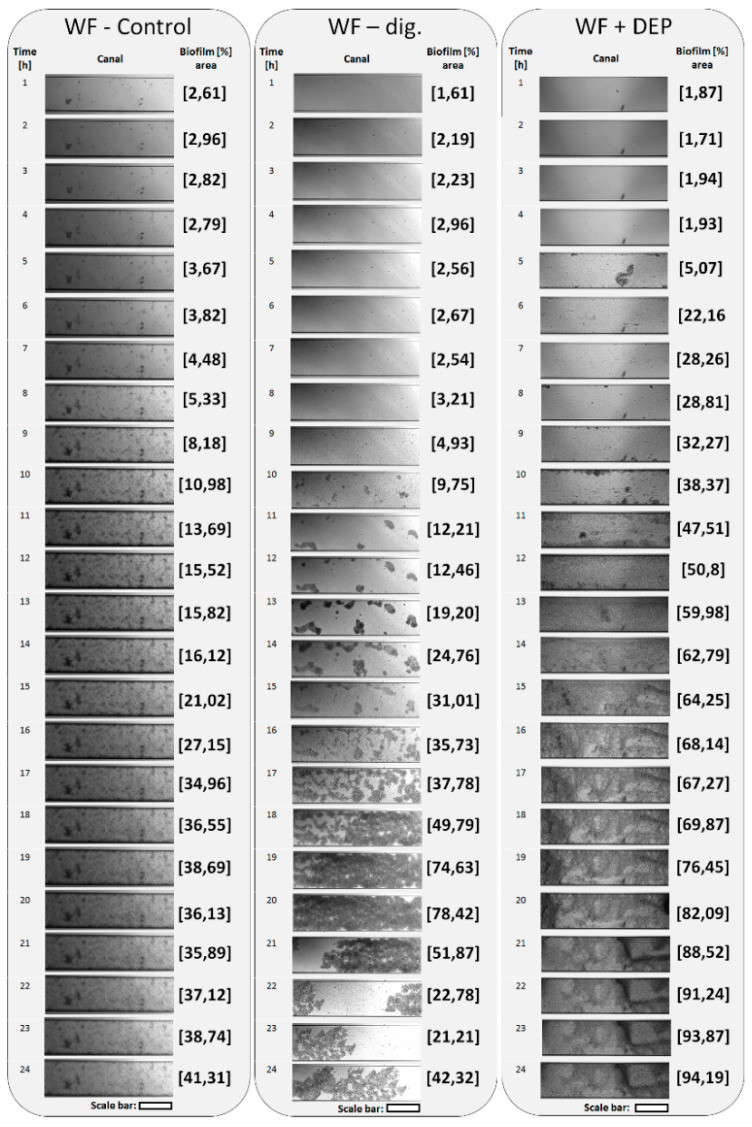
Imaging of the channel cross section microfluidic system for microbiome of bioreactor samples during 24 h of incubation at flow conditions (0.5 dyn cm^2^). Biofilms formed by microorganisms were visualised using differential interference contrast (DIC); scale bar = 100 µm.

The results of the analysis of the ability to adhere and form biofilm over time, in an anaerobic environment, showed that all test samples, taken from the bioreactor, were able to form biofilm within 24 h (see Figure 9). Relatively faster adhesion was observed for the WF-control sample, where already in the first 5 h a high level of adhered cells was recorded, in comparison to the other test samples. Thereafter, the surface area of adhered cells and biofilm increased steadily to reach more than 41% of the channel volume under flow conditions during the 24-h incubation. Detailed NGS analysis of the sample showed that it had the highest microbial biodiversity (28 common taxa and 20 completely separate ones, representing individual characteristics of the sample), which may have been particularly important for the first stages of adhesion (see Venn diagram, Figure 5). However, the fill rate of just over 41% may reflect the fact that, despite the high biodiversity of the microorganisms, they are not able to form a high biomass together, which in turn may be due to some antagonism between populations of these taxa [65]. For example, species in a biofilm may compete for nutritional resources or alternatively, they may be better able to utilise nutrients and remain more resilient to harsh conditions. As observed by Elias and Banin (2011), there are also species in nature that do not normally form single-species biofilms but may be involved in the formation of mixed-species biofilms [66].

In the case of another sample, WF-dig., taken from the bioreactor at the final stage of the fermentation process, a visible increase in the number of adhered cells was observed only after 10 h (almost 10%). After this time, the amount of biofilm on the canal surface increased steadily, reaching its maximum at 20 h (more than 78%). Thereafter, the amount of biofilm decreased, finally reaching 42%. This issue represents a limitation of flow systems, as dense biofilm formation may contribute to increased flow in the canal and/or its subsequent clogging. 

When conducting an analysis of biofilm formation in continuous flow, it is important to consider that in a fluid environment, biofilm formation is closely related to relevant flow field parameters such as shear stress, secondary flow and Reynolds number. Recently, Liu et al. (2022) presented the results of the study on the influence of microfluidic channel geometry on *Bacillus subtilis* biofilm formation [67]. This author demonstrated that both shear stress and secondary flow play an important role in biofilm adhesion at the initial stage. Shear stress determines whether the biofilm adheres; secondary flow, thereafter, determines the rate of adhesion. The experiment mentioned above also proved that once the biofilm is formed, its structure changes from loose to dense, with a 20-fold increase in adhesion, which is of practical importance.

It should be added that in this paper, NGS analysis showed a decrease in biodiversity of the WF-dig. sample in comparison to the WF-control sample; 28 common taxa were observed in this case and only six were found exclusively in this sample (Figure 5). On this basis it can be concluded that the methane fermentation process probably contributed to the formation of a less diverse, but much more stable and synergistic microbiome, producing an efficient biofilm (Figure 4 and Figure 9). As noted in her paper by Besemer (2015) the stable juxtaposition of microbial cells renders biofilms coordinated functional consortia, which makes them more efficient than mixed communities [68].

The results analysis for the sample WF + DEP (with the addition of the carrier and taken at the final stage of fermentation), showed the fastest microbial adhesion capacity, already after 5 h, reaching just over 5% (see Figure 9). Moreover, from the 10th hour of the experiment, quite visible biofilm agglomerates were observed forming, occupying more than 38% of the canal surface. It is noteworthy that many numerous but small adhesive foci were observed, which led to an almost complete filling of the canal (94%) by biomass within 24 h. This phenomenon should be explained by the fact that microorganisms associated with the matrix, in this case with the diatomaceous earth/peat system, show a much better ability to adhere—to the surface of the glass plate, under conditions of dynamic flow of the substrate, e.g., due to intensified synthesis of numerous adhesive proteins and stronger connection with a given abiotic surface [69]. The selection of taxa, performed on the basis of NGS analysis, may also be important, which shows that a decrease in their number results in better dynamics and structure of the biofilm (Figure 5). These observations also correlate with the total bacterial count results of the fermented sample with the addition of the carrier (see Figure 3).

### 3.5. Visualisation of Microbiomes from Bioreactor Samples

**Figure 10:**
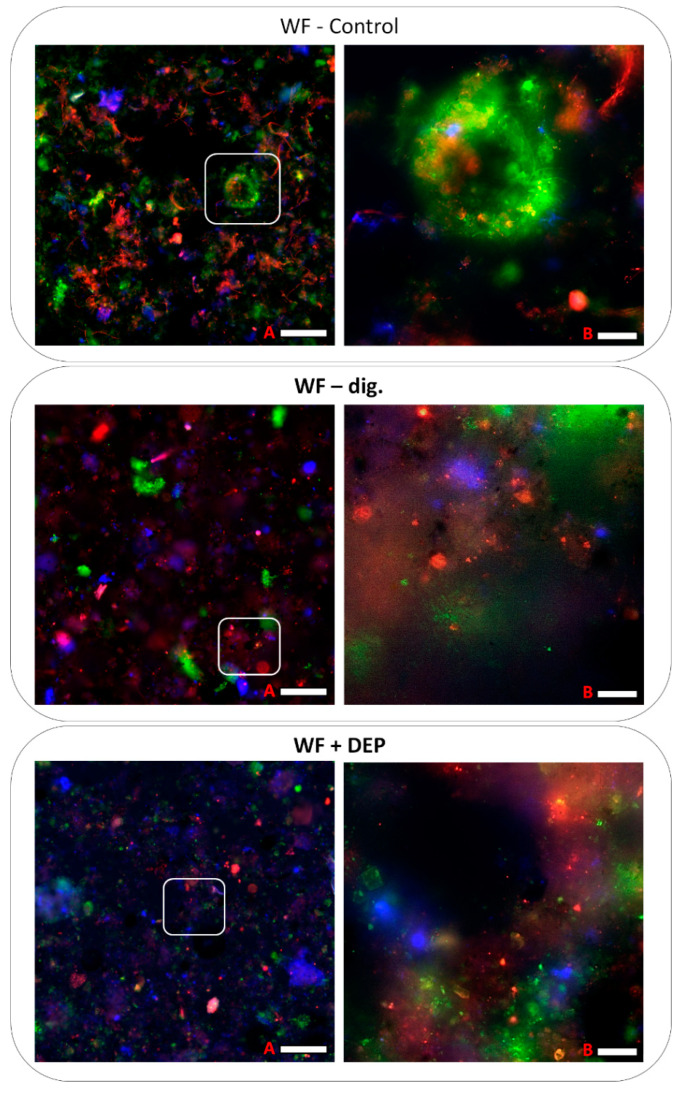
Visualisation of biofilms produced by the microbiome of raw research samples from the bioreactor. Green fluorescence—live cells; red fluorescence—dead cells; blue or pink fluorescence—carrier with bacteria. Scale bar A = 100 µm (left side) or B = 20 µm.

This visualisation provides an excellent research summary of the work, showing the complex biodiversity and structure of the biofilms of the samples studied (see Figure 10). Live/dead staining was used to determine the viability of the biofilms. Microscopic analysis of the WF-control sample revealed a large variety of spatial forms of biofilms as well as varied morphology. During imaging it was observed that the bacteria form very diverse consortia, consisting of anaerobic and aerobic bacteria (archaeons of the genus *Methanolinea*, but also *Brevundimonas*, *Lacipirellula*, *Myroides*, *Phyciococcus* and the genus of *Comamonas*, see Figure 5).

The predominant morphological forms in the accompanying images are filamentous, spindle-shaped, small coccobacilli as well as small bacilli and numerous cocci, often surrounded by large amounts of exopolysaccharides. Such a high diversity of bacterial cell morphology is a characteristic feature of sewage sludge [70].

In the case of the WF-dig. sample (coming from the final phase of AD, without carrier), a decrease in morphological biodiversity and a lower diversity of spatial forms of biofilms was observed. What is noteworthy here is the appearance of a biofilm, probably formed from granules or very small granulobacteria surrounded by a rich extracellular matrix. Images of the sample after fermentation with the carrier (WF + DEP) are very similar but characterised by even less diversity despite the visible (blue luminescent) carrier materials.

Moreover, it is possible to observe numerous adhered cocci on the carriers, which may confirm that they may be a source of increased bacterial biomass. It is essential to emphasise that the above material is best followed together with the NGS results (Figure 5), which shines full light on the content of the taxa present and at the same time a complete insight into the microbial diversity of the sample.

### 3.6. Summary and a Look to the Future

The presentation of the results of the metagenomic analysis in the different reference systems (see Figure 4, Figure 5, Figure 6, Figure 7 and Figure 8 and Table 2), clearly indicated the key directions of qualitative and quantitative changes in the microbiome that occurred (comparison of WF-control with WF-dig. and WF + DEP) under the influence of the anaerobic degradation of organic matter, included in the samples, and by the action of the DEP carrier (differences between WF-dig. and WF + DEP). The results of the NGS analysis, confirmed by studies of the cells’ ability to adhere and form biofilm over time, proved a reduction in the number of taxa in the degraded samples, with a simultaneous increase in the efficiency of biofilm formation. Noteworthy in this respect are the following facts: as a result of the modification of the microbiome, the samples were dominated by clusters of Firmicutes, showing an ability to form spore forms, and Synergistetes, which, respectively, appeared in the place of Actinobacteria and Proteobacteria. The changes taking place in the microbiome are determined by constant and changing environmental conditions during the process, among which the anaerobic conditions, the environmental reaction, the temperature and the depleting medium, as well as additives, in this case cell carriers [18,71]. As confirmed in the recent work by Cayetano et al., (2022), different carrier materials strongly induce the dynamics of the dominant microbial population in each system [5]. Immobilised, selected high-metabolism and high-survival rate bacterial cells form a much more stable and synergistic microbiome (here composed of Firmicutes and Synergistetes phylum), efficiently producing a biofilm, which was the case in the experiment carried out. Under the optimal conditions obtained in this case, the recovery of biogas including methane occurred efficiently until the nutrients were depleted (see Figure 3), which had a significant impact on the efficiency of the AD process.

On the other hand, the recorded differences in bacterial structure and abundance at genus level between the unsupplemented sample and the DEP-supplemented sample highlighted the dominance of the genus *Syntrophomonas*, particularly in the DEP-supplemented sample. It is, as mentioned earlier, a genus that plays an important role in LCFA (long chain fatty acid) oxidation and degradation of lower organic acids, growing syntrophically with hydrogenotrophic methanogens [72].

At this point, it is worth recalling that the process of anaerobic catabolism, involving the breakdown of substance molecules into smaller ones, requires close cooperation between two or more microorganisms of different species and is referred to as syntrophy. The interaction between fermentative bacteria and methanogenic archaea to overcome the thermodynamic barrier [73] of lowering the activation energy and consequently increasing the reaction rate in the first step of the breakdown of organic compounds (e.g.,: fatty acids, alcohols, or aromatic compounds) is one of several examples of syntrophy in the environment. 

The presence of *Syntrophomonas* (propionate and butyrate degraders) and Synergistetes (syntrophic acetate oxidisers), as in the present study, might be a sign of well acetogenicity in anaerobic bioreactors. Higher content of *Syntrophomonas* in WF + DEP, compared to WF-dig. indicates better conditions for its development and more active participation in biomass conversion—both in the “cutting” of long carbon chains (acidogenic phase) and degradation of subsequent intermediates, such as propionic and butyric acids (acetogenic phase), which resulted in a greater possibility of fast and efficient recovery of methane as an energy carrier (methanogenic phase) [13]. As can be seen from the above, the rate and directions of change within the metapopulation have a significant impact on the performance of the AD process. On the other hand, the use of the DEP carrier, whose action in the presented experiment resulted in a targeted and selective modification of the microbiome in AD, and consequently in the development of cells whose enzymes effectively catalysed the critical and decisive transformation process, is an appropriate solution to the problems of AD implementation mentioned in the introduction.

The diverse microbial syntrophisms involved in anaerobic digestion are generally mediated by electron transfer [74]. Currently, researchers are interested in the carriers that mediate the electron transfer involved in methanogenesis. It has been suggested that the promotion of surface contact and improvement of interspecies electron transport have a major impact on the results of anaerobic treatment [5]. Modern spectroscopy techniques provide important information on the composition and structural organisation of the biofilm which can be useful in elucidating the added function of this special layer of microbial cells. Also, for a few years now, the addition of iron based conductive materials, represented by magnetite, have been intensively studied in AD to help establish the DIET (direct interspecies electron transfer) pathway [75,76].

Referring to such an important issue for the AD process, it is worth noting the great advantage of both diatomite and peat which, due to their structure and chemical composition, are materials with high conductivity and can successfully act as electron transporters [77]. The clearly positive effect of the addition of the DEP hybrid (ultimately increased methane production), consisted simultaneously of three of its actions: (i) targeted changes in the microbiome; selectivity—promoting the growth of key bacterial genera (ii) increased activity of the transformation biocatalyst, as a result of improved environmental conditions, and (iii) increased electron transfer efficiency, due to, among other things, the good conductivity of the carrier. These issues should be subject to further research into the mechanisms by which the DEP system affects the microbiome of anaerobic digestion environments.

## 4. Conclusions

The analysis of selected physicochemical properties of the DEP carrier used in this study showed that it is a suitable microbial additive for both mesophilic and thermophilic AD, mainly due to its porous microstructure, highly developed specific surface area (due to diatomite, 36.0 m^2^/g), high thermal stability and compatibility.

The metagenomic analysis results presented in different reference systems proved that both the fermentation process and the addition of the DEP system contribute to the modification of the bacterial microbiome in anaerobic bioreactors. The fermentation process noticeably altered the microbiome, reducing its biodiversity, as observed in the decrease in the number of taxa in samples WF-dig. and WF + DEP. On the other hand, the results of the BioFlux microfluidic flow system method, showed that a decrease in the number of taxa in the digested samples and the addition of DEP promoted microbial adhesion in the microfluidic system and the formation of a stable biofilm. The greatest capacity for adhesion and biofilm formation was observed in the case of the WF + DEP sample (94% biomass filling of the canal was achieved within 24 h), compared to the other test samples. At the same time, an intensified multiplication of anaerobic bacteria was recorded in the system with the addition of DEP, up to the last term of the process, indicating an increase in their viability through the effective and selective effect of the matrix and constant access to the medium.

The assessment of quantitative and qualitative changes in genetic diversity of bacterial communities identified the most important directions for modification of the bacterial microbiome. The proportions of each cluster shifted during the process: the most abundant cluster in the WF-control sample was Actinobacteria, while Firmicutes were the dominant cluster in the WF-dig. and in WF + DEP. Significant statistical differences in bacterial structure and abundance at the genus level were observed between the ferment without and with the addition of the DEP carrier. These variants were dominated by the genus *Syntrophomonas* as well as *Clostridium sensu stricto*. The highest percentage of its sequence was recorded in the object with the addition of the carrier (WF + DEP). The increased amounts and activity of bacteria of the *Syntrophomonas* genus, may have been a key determinant in improving the efficiency of CH_4_ production in the carrier sample.

## Figures and Tables

**Table 1:** Composition and selected properties of the feedstock/inoculum batches.

Batches	WF (g)	Carrier (g)	Inoculum (g)	pH	TS (%)	VS (%)
WF-control	9.8	-	830.2	7.15	4.00	72.50
WF + DEP	9.8	20.0	830.3	7.03	3.96	70.62

**Table 2:** The available information about selected, most abundant unclassified sequences, based on the NCBI database.

Unclassified Symbol (in This Research)	NCBI Accession Numbers (% of Sequence Identity)	Source/Environment	References (If Available)	Closest Relative
unclassified_0010	EF059533 (97.2%)	PCB-dechlorinating enrichment culture	Bedard et al. (2007) [46]	*Sedimentibacter* sp.
	AY766467 (96.5%)	Anaerobic coculture enriched from a hexachlorocyclohexane (HCH) polluted soil.	Wim van Doesburg et al. (2005) [47]	*Sedimentibacter* sp.
unclassified_0015	MK143173 (98.8%)	Algae (Iceland)	Costa et al. (2019) [48]	*Knoellia* sp.
	KX256211 (98.8%)	Eastern Mediterranean sea sediment	Gärtner et al. (2016) [49]	*Intrasporangium* sp.
unclassified_0047	NR_041354 (97%)	Thermophilic digester sludge, methanogenic propionate-degrading consortia	Yamada et al. (2007) [50]	*Bellilinea caldifistulae*
	KX261406 (93.8%)	Sludge and beet sugar industrial wastewater	-	*Levilinea saccharolytica*
unclassified_0018	AB021325 (98%)	Activated sludge with phenol as the sole carbon source	Watanebe et al. (1999) [51]	Uncultured/unclassified
	JQ899231 (97.5%)	Marine soil sediment	-	*Streptomyces aomiensis*
unclassified_054	AB529706 (98%)	Rhizoplane	Tanaka et al. (2012) [52]	Uncultured/unclassified
	HM124367 (96.8%)	Lake sediment	-	*Hyphomicrobium* sp.
unclassified_0012	MN826598 (98.52%)	Rhizosphere in fertilised and degraded soils	-	*Janibacter limosus*
unclassified_0041	KX876303 (100%)	Dark fermentation reactor	Chatellard et al. (2016) [45]	Uncultured/unclassified
unclassified_0059	KM675947 (100%)	Rhizosphere soil	Arulmani and Jebakumar (2015) [53]	*Hydrogenophaga* sp.
unclassified_0062	MH553022 (99.76%)	Bioreactor	-	Alcaligenaceae bacterium
	HQ670757 (99.53%)	Sugarcane molasses-based distillery	-	*Pusillimonas* sp.
unclassified_0021	MG910712 (100%)	Bioreactor	-	Uncultured/unclassified
	AB997768 (100%)	Sludge from full scale anaerobic digester	-	Uncultured/unclassified
unclassified_0057	MG910621 (100%)	Bioreactor	-	Uncultured/unclassified
	CU922732 (100%)	Wastewater sludge	Rivière et al. (2009) [54]	Uncultured Deltaproteobacteria

## Data Availability

Not applicable.
